# Cohort profile: Improved Pregnancy Outcomes via Early Detection (IMPROvED), an International Multicentre Prospective Cohort

**DOI:** 10.12688/hrbopenres.13812.3

**Published:** 2024-06-24

**Authors:** Gillian M. Maher, Louise C. Kenny, Kate Navaratnam, Zarko Alfirevic, Darina Sheehan, Philip N. Baker, Christian Gluud, Robin Tuytten, Marius Kublickas, Boel Niklasson, Johannes J. Duvekot, Caroline B. van den Berg, Pensee Wu, Karolina Kublickiene, Fergus P. McCarthy, Ali S. Khashan

**Affiliations:** 1 INFANT Research Centre, University College Cork, Cork, T12YE02, Ireland; 2 School of Public Health, University College Cork, Cork, T12XF62, Ireland; 3 Department of Women’s and Children’s Health, Faculty of Health and Life Sciences, University of Liverpool, Liverpool, L693BX, UK; 4 College of Life Sciences, University of Leicester, Leicester, LE17RH, UK; 5 Copenhagen Trial Unit, Centre for Clinical Intervention Research, Copenhagen University Hospital - Rigshospitalet, Copenhagen, The Capital Region, Copenhagen, DK2200, Denmark; 6 Department of Regional Health Research, The Faculty of Health Sciences, University of Southern Denmark, Odense, DK5230, Denmark; 7 Metabolomic Diagnostics, Cork, T45YX04, Ireland; 8 Department of Fetal Medicine, Karolinska University Hospital, Stockholm, SE17176, Sweden; 9 Department of Nursing Science, Sophiahemmet University, Stockholm, SE11486, Sweden; 10 Department of Obstetrics and Gynecology, Division of Obstetrics and Prenatal Medicine, Erasmus MC, University Medical Center Rotterdam, Rotterdam, 3015GD, The Netherlands; 11 School of Medicine, Keele University, Staffordshire, ST55BG, UK; 12 Division of Renal Medicine, CLINTEC, Karolinska Institutet, Stockholm, SE14152, Sweden; 13 Department of Obstetrics and Gynaecology, University College Cork, Cork, T12YE02, Ireland

**Keywords:** Cohort profile, preeclampsia, biobank, clinical data

## Abstract

**Background:**

Improved Pregnancy Outcomes via Early Detection (IMPROvED) is a multi-centre, European phase IIa clinical study. The primary aim of IMPROvED is to enable the assessment and refinement of innovative prototype preeclampsia risk assessment tests based on emerging biomarker technologies. Here we describe IMPROvED’s profile and invite researchers to collaborate.

**Methods:**

A total of 4,038 low-risk nulliparous singleton pregnancies were recruited from maternity units in Ireland (N=1,501), United Kingdom (N=1,108), The Netherlands (N=810), and Sweden (N=619) between November 2013 to August 2017. Participants were interviewed by a research midwife at ~11 weeks (optional visit), ~15 weeks, ~20 weeks, ~34 weeks’ gestation (optional visit), and postpartum (within 72-hours following delivery).

**Findings to date:**

Clinical data included information on maternal sociodemographic, medical history, and lifestyle factors collected at ~15 weeks’ gestation, and maternal measurements, collected at each study visit. Biobank samples included blood, urine, and hair collected at each study visit throughout pregnancy in all units plus umbilical cord/blood samples collected at birth in Ireland and Sweden. A total of 74.0% (N=2,922) had an uncomplicated pregnancy, 3.1% (N=122) developed preeclampsia, 3.6% (N=143) had a spontaneous preterm birth, and 10.5% (N=416) had a small for gestational age baby. We evaluated a panel of metabolite biomarkers and a panel of protein biomarkers at 15 weeks and 20 weeks’ gestation for preeclampsia risk assessment. Their translation into tests with clinical application, as conducted by commercial entities, was hampered by technical issues and changes in test requirements. Work on the panel of proteins was abandoned, while work on the use of metabolite biomarkers for preeclampsia risk assessment is ongoing.

**Future plans:**

In accordance with the original goals of the IMPROvED study, the data and biobank are now available for international collaboration to conduct high quality research into the cause and prevention of adverse pregnancy outcomes.

## Introduction

Preeclampsia is one of the leading causes of maternal morbidity and mortality in Europe ^1^. It is defined as gestational hypertension (systolic blood pressure (BP) ≥140 mmHg and/or diastolic BP ≥90 mmHg (Korotkoff V)) on at least two occasions 4 hours apart after 20 weeks’ gestation, but before the onset of labour, or postpartum systolic BP ≥140 mmHg and/or diastolic BP ≥90 mmHg on at least two occasions 4 hours apart with proteinuria (≥300 mg/24 hours, or spot urine protein:creatinine ratio ≥30 mg/mmol creatinine, or urine dipstick protein >/= ++) ^2^. Preeclampsia affects up to 5% of all pregnancies and can lead to acute problems in the liver, kidneys, brain, and the clotting system, and is associated with an increased risk of cardiovascular and metabolic diseases later in life ^2– 5^.

Improved Pregnancy Outcomes via Early Detection (IMPROvED) is a multi-centre, European phase IIa clinical study (ClinicalTrials.gov Identifier: NCT01891240). The IMPROvED Consortium was set up to develop a clinically useful screening test for preeclampsia to assist in offering targeted surveillance or preventative strategies. To achieve this, a high calibre pregnancy biobank augmented with well-curated patient and clinical information was required to evaluate panels of metabolomic and proteomic biomarkers, which were previously shown to be predictive of preeclampsia ^6, 7^. In accordance with the objectives of the IMPROvED project ^2^, a prospective study was set up by the clinical collaborators within the IMPROvED Consortium. First-time mothers across participating maternity units in Republic of Ireland, United Kingdom, The Netherlands, and Sweden were invited early in pregnancy to participate in the IMPROvED study and to consent to the taking, and biobanking, of biospecimens at defined times during their pregnancy for analysis of (preeclampsia) biomarkers. Detailed demographic and clinical data were collected from study participants in each participating maternity unit, and maternal measurements were performed at multiple time points across their pregnancies. Furthermore, to maximise the utility of the IMPROvED cohort, detailed data on pregnancy outcomes, including key outcomes of interest such as spontaneous preterm birth ( *i.e.*, delivery <37+0 weeks’ gestation) and small for gestational age (SGA) ( *i.e.*, birthweight <10th customised centile) were collected ^2^.

In parallel to recruitment taking place, the IMPROvED project foresaw for translational research to be conducted at the commercial partners. The planned research primarily focused on replacing the biomarker measurement technology as used in identifying the respective metabolite-, and protein-biomarker panels with (commercially viable) biomarker measurement technology suitable for application in clinical laboratories. The envisioned biomarker tests were labelled MetTest and ProTest, respectively ^2^.

The aim of this cohort profile is to firstly provide a detailed description of the IMPROvED cohort, including data collection and follow-up procedures; secondly an update on findings reported thus far by the IMPROvED Consortium regarding the goals and objectives of the IMPROvED project, and thirdly details on how the scientific community can access IMPROvED data for research projects.

## Methods

### Cohort setting, location, and key dates

The IMPROvED cohort contains hospital-based maternity data from Republic of Ireland (University College Cork), United Kingdom (Keele University, University of Liverpool, and University Centre Shrewsbury), The Netherlands (Erasmus MC, University Medical Center, Rotterdam), and Sweden (Karolinska Institutet, Stockholm). Recruitment took place between 29th November 2013 and 3rd August 2017. While work on the use of metabolite biomarkers for preeclampsia risk assessment is ongoing, the data and biobank are now available for international collaboration to conduct high quality research into the cause and prevention of adverse pregnancy outcomes.

All centres obtained ethical approval for the IMPROvED consortium project from their respective ethic committees (Clinical Research Ethics Committee of the Cork Teaching Hospitals: ECM5(3)06/08/13 in August 2013; West Midlands - Solihull Research Ethics Committee: 13/WM/0268 in July 2013; Medical Ethics Committee Erasmus MC of Rotterdam: NL44426.078.13 in July 2013, and EPN – Stockholm Regional Ethics Review Board: 2013-306-31-2 in April 2013). Informed consent was signed by participants at the first study visit (11+0 to 13+6 weeks’ gestation).

### Eligibility criteria and inclusion

Eligibility criteria included females aged 16 years or older, nulliparous, singleton pregnancy, and signed informed consent. Full exclusion criteria have been published previously ^2^ and included the following: unsure of last menstrual period and unwilling to have ultrasound scan at ≤20 weeks’ gestation; ≥3 miscarriages; ≥3 terminations; known or suspected major foetal anomaly/abnormal karyotype; essential hypertension treated pre-pregnancy; moderate-severe hypertension at booking (BP >160/100 mmHg); diabetes mellitus; renal disease; systemic lupus erythematosus; anti-phospholipid syndrome; sickle cell disease; HIV positive; major uterine anomaly; cervical suture *in situ*; knife cone biopsy; long-term glucocorticosteroids; treatment with low-dose aspirin; or treatment with heparin/low molecular weight heparin ^2^.

**Figure 1.**
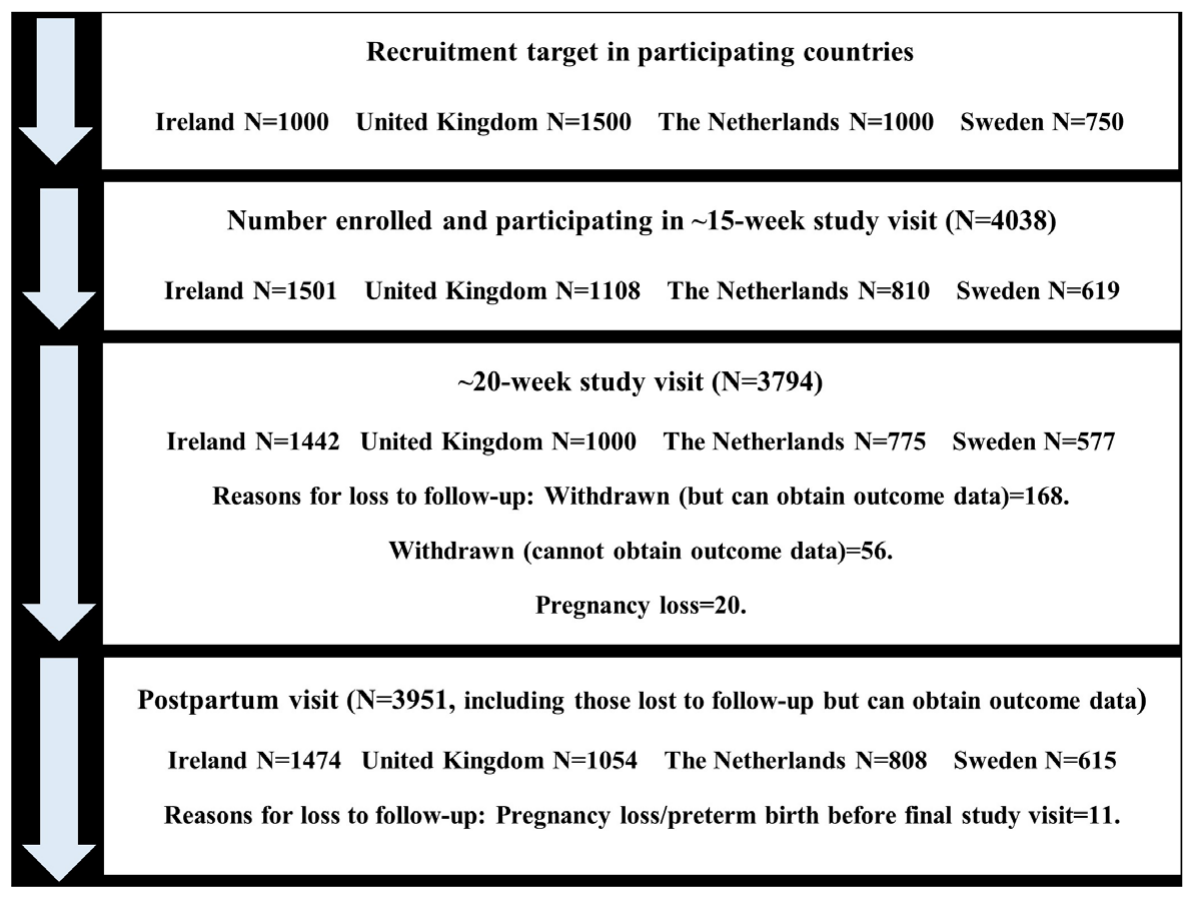
Flow diagram of study participants for main study visits in the IMPROvED cohort.

Initially recruitment was planned in five European countries with the following recruitment targets: Republic of Ireland (N=1,000), United Kingdom (N=1,500), The Netherlands N=1,000, Sweden (N=750), and Germany (N=750) ^2^. However, the study was not feasible at the German site, and they subsequently withdrew from the study at an early stage ^8^. The following samples were enrolled in each remaining countries: Republic of Ireland N=1,501; United Kingdom N=1,108; The Netherlands N=810, and Sweden N=619 ( Figure 1 and Table 1).

**Table 1.** Baseline characteristics of study participants enrolled in the IMPROvED cohort by country (N=4,038).

*Characteristics*	*Total* *N=4038*	*Republic of* * Ireland* *N=1501*	*United * *Kingdom * *N=1108*	*The * *Netherlands * *N=810*	*Sweden* *N=619*
*Maternal age*					
18–27 years	1214 (30.0)	324 (21.6)	498 (45.0)	216 (26.7)	176 (28.4)
28–37 years	2692 (66.7)	1123 (74.8)	591 (53.3)	557 (68.7)	421 (68.0)
38–47 years	132 (3.3)	54 (3.6)	19 (1.7)	37 (4.6)	22 (3.6)
*Maternal body mass index*					
Underweight/normal weight	2377 (59.0)	837 (55.8)	575 (52.0)	547 (67.7)	418 (67.6)
Overweight	1175 (29.1)	485 (32.4)	349 (31.5)	184 (22.8)	157 (25.4)
Obese	480 (11.9)	177 (11.8)	183 (16.5)	77 (9.5)	43 (7.0)
Missing data	6	2	1	2	1
*Education*					
Third level	2457 (60.9)	1056 (70.4)	573 (51.7)	371 (45.8)	457 (73.8)
Less than third level	1581 (39.1)	445 (29.6)	535 (48.3)	439 (54.2)	162 (26.2)
*Employment status*					
In paid employment	3609 (89.4)	1341 (89.3)	992 (89.5)	709 (87.5)	567 (91.6)
Not in paid employment	429 (10.6)	160 (10.7)	116 (10.5)	101 (12.5)	52 (8.4)
*Relationship status*					
Married/stable relationship	3823 (94.7)	1400 (93.3)	1044 (94.2)	775 (95.7)	604 (97.6)
Single/separated/divorced	215 (5.3)	101 (6.7)	64 (5.8)	35 (4.3)	15 (2.4)
*Maternal smoking before/during pregnancy*					
Non-smoker	3139 (77.8)	1093 (72.8)	882 (79.6)	644 (79.5)	520 (84.0)
Quit before first study visit	650 (16.1)	292 (19.5)	130 (11.7)	137 (16.9)	91 (14.7)
Smoked at time of first study visit	248 (6.1)	116 (7.7)	95 (8.7)	29 (3.6)	<10
Missing data	1	0	1	0	0
*Maternal alcohol consumption before/during pregnancy*					
Non-drinker	968 (24.0)	133 (8.9)	433 (39.1)	279 (34.4)	123 (19.9)
Quit before first study visit	3008 (74.5)	1330 (88.6)	657 (59.3)	528 (65.2)	493 (79.6)
Drank alcohol at time of first study visit	61 (1.5)	38 (2.5)	17 (1.5)	<10	<10
Missing data	1	0	1	0	0

N (%) for categorical variables.

### Data collection and follow-up

Only those who consented to sampling procedures at the second (~15 weeks’ gestation) and third (~20 weeks’ gestation) time-points were eligible for recruitment. While participation at the first (~11 weeks’ gestation) and fourth (~34 weeks’ gestation) time-points were desirable, these were not mandatory. Informed written consent was obtained from all participants at their first study visit. Participants were interviewed by a research midwife at each sampling time-point and all data, including data on storage details of specimens, were entered directly into the IMPROvED database. Participants were instructed to contact the research midwife if delivery occurred before the final study visit or if they developed preeclampsia, had a spontaneous preterm birth or delivered a small for gestational age (SGA) baby ^2^.

*First sampling (optional visit):* The first study visit took place at 11+0 to 13+6 weeks’ gestation. Maternal measurements were performed for height, weight, blood pressure, pulse, urinary protein, and blood glucose for a maximum of 1,076 participants. Specimens including non-fasting 30 ml blood, 10 ml mid-stream sample of urine, and sample of hair were also collected for 1,076 participants.

*Second sampling:* The second study visit took place at 14+0 to 16+6 weeks’ gestation, resulting in a total sample size of 4,038 participants (including the 1,076 participants from the first non-mandatory study visit). If maternal measurements were not taken at the first study visit, these were taken at the second study visit. Specimens including non-fasting 30 ml blood (n=3,992), 10 ml mid-stream sample of urine (n=3,992), and sample of hair (n=3,081) were also collected. Information on demographics, current pregnancy details, and lifestyle factors were collected during this visit through interview with a research midwife. Demographic information included maternal age, marital status, ethnicity, country of birth, education, occupation, living situation, household income and type of maternity care. Current pregnancy details included information such as gravidity and history of pregnancy complications, for example, infertility, hypertensive disorders of pregnancy and stillbirth, as well as any medical conditions. Lifestyle factors included data on smoking, alcohol use, as well as multivitamin use during pre-pregnancy, during the first trimester, and by the first study visit ( Table 2).

**Table 2.** Prenatal and postpartum data available for IMPROvED participants.

*Time-points of Sampling Procedures*						
Biobank data	*~11 weeks*	*~15 weeks*	*~20 weeks*	*~34 weeks*	*At birth*	*Postpartum* *(within 72 hours)*
Non-fasting 30ml blood	✓	✓	✓	✓		
10ml mid-stream sample of urine	✓	✓	✓	✓		
Sample of hair	✓	✓	✓	✓		
Umbilical cord blood ^a^					✓	
Umbilical cord sample ^a^					✓	
Placental samples					✓	
Maternal measurements						
Height ^b^	✓	✓				
Weight ^b^	✓	✓	✓	✓		
Blood pressure ^b^	✓	✓	✓	✓		
Pulse ^b^	✓	✓	✓	✓		
Urinary protein ^b^	✓	✓	✓	✓		
Blood glucose ^b^	✓	✓	✓	✓		✓
Demographics and lifestyle factors						
Maternal age	✓					
Marital status		✓				
Ethnicity		✓				
Country of birth		✓				
Education		✓				
Occupation		✓				
Living situation		✓				
Household income		✓				
Type of maternity care		✓				
Smoking		✓				
Alcohol/Drug use		✓				
Multivitamin use		✓				
Pregnancy, delivery, and infant data						
History of pregnancy complications		✓				
Medical conditions		✓				
Gravidity		✓				
Mode of delivery						✓
Infant sex						✓
Preeclampsia						✓
Gestational hypertension						✓
Placental abruption						✓
Rupture of membranes						✓
Birthweight						✓
Gestational age						✓
Baby’s length						✓
Baby’s head circumference						✓
Estimated blood loss						✓
Apgar scores						✓
Severe neonatal morbidity						✓
Admitted to neonatal unit						✓
Reason for neonatal unit admission						✓
Paternal data						
Age ^c^	✓	✓	✓	✓	✓	✓
Blood sample for DNA analysis ^c^	✓	✓	✓	✓	✓	✓

^a^Taken at centres in Republic of Ireland and Sweden, only. ^b^If measurements were not taken at the first study visit, these were taken at the second study visit. ^c^Taken at centre in Republic of Ireland only, and at any one visit or by extra appointment.

*Third sampling:* The third study visit took place at 19+0 to 21+6 weeks’ gestation. Maternal measurements were performed for weight, blood pressure, pulse, urinary protein, and blood glucose for a maximum of 3,794 participants. Specimens including non-fasting 30 ml blood (n=3,794), 10 ml mid-stream sample of urine (n=3,794), and sample of hair (n=3,083) were also collected

*Fourth sampling (optional visit):* The fourth study visit took place at 32+0 to 34+6 weeks’ gestation. Maternal measurements were performed for weight, blood pressure, pulse, urinary protein, and blood glucose for a maximum 1,313 participants. Specimens including non-fasting 30 ml blood (n=1,313), 10 ml mid-stream sample of urine (n=1,313), and sample of hair (n=1,024) were also collected.

*At birth (optional visit):* Placental samples (n=59) were taken shortly after delivery. At centres in Republic of Ireland and Sweden, blood from the umbilical cord and a sample of the cord itself were taken for 974 and 41 participants, respectively.

*Postpartum:* Within 72 hours following delivery, information about the pregnancy, delivery and the baby were obtained by a research midwife through interview/reviewing medical records. The total sample size at this visit was 3,951 participants (including those lost to follow-up but for whom outcome data could be obtained from medical records, N=168). This data included, but was not limited to, information on mode of delivery, infant sex, hypertensive disorders of pregnancy, placental abruption, rupture of membranes, birthweight and gestational age of baby, Apgar scores at 1 and 5 minutes, any severe neonatal morbidity, whether infant was admitted to neonatal unit as well as reason for neonatal unit admission ( Table 2). If possible, the baby’s measurements were also taken at this time. If not, these were obtained from medical records. All information was confirmed by reviewing medical records. Information about complications of pregnancy, including the primary outcomes were also recorded. Any participant who developed preeclampsia, experienced spontaneous preterm birth, or delivered an SGA baby had detailed clinical, laboratory, and outcome data collected ^2^.

### Database and biobank development

An IMPROvED customised clinical data and biobank management database was developed in Sweden. This database was specifically designed for data management in clinical trials and cohort studies. Comprehensive clinical data, blood, urine, and hair samples were collected and recorded in this database at each study visit.

In addition, IMPROvED established a high calibre pregnancy biobank containing samples from participants at each study visit. The IMPROvED pregnancy biobank is housed at University of Cork, Ireland. Both the epidemiological data and biobank samples can be used by the scientific community to conduct high quality research into maternal and child health.

### Data analysis

All descriptive statistics for the current study were performed using Stata MP 14.2 (RRID:SCR_012763) (free alternative, RStudio).

## Results

### Baseline characteristics

Baseline characteristics of study participants are outlined in Table 1. Briefly, most participants were in the 28–37 years age bracket (66.7% overall). The United Kingdom had a higher proportion (45.0%) of younger participants enrolled ( *i.e.*, 18 to 27 year-olds) compared to other countries (30.0% overall). A slightly higher proportion of obese participants were enrolled in the United Kingdom (16.5%), while a lower proportion were enrolled in Sweden (7.0%), compared to 11.9% overall. The majority of participants had a third level of education (60.9% overall). However, this was less pronounced in the United Kingdom (51.7%) and The Netherlands (45.8%). Most participants were in paid employment across all countries (89.4% overall). Similarly, most participants were married or in a stable relationship (94.7% overall). The majority of participants were non-smokers (77.8% overall). However, a slightly higher proportion of participants smoked at time of first study visit in Republic of Ireland (7.7%) and the United Kingdom (8.7%) compared to 6.1% overall. There were fewer non-drinkers of alcohol (defined as zero alcohol intake in the three months prior to pregnancy) enrolled in Republic of Ireland (8.9%) and Sweden (19.9%) compared to the United Kingdom (39.1%) and The Netherlands (34.4%). Overall, 74.5% stopped drinking alcohol before the first visit, while 2.5% of participants in the Republic of Ireland were still drinking alcohol at time of first study visit compared to 1.5% overall. Missing data was <1% at baseline.

### Findings to date

Data on 87 participants were lost to follow-up between enrolment and the final study visit (within 72 hours following delivery), resulting in 3,951 participants with postpartum outcome data. Of these, 74.0% (N=2922) had an uncomplicated pregnancy. Similar to estimates reported elsewhere ^5, 9^, approximately 3.0% (N=122) of participants developed the primary study outcome of preeclampsia. This is subdivided by country as follows: Republic of Ireland N=57 (3.9%); United Kingdom N=30 (2.9%); The Netherlands N=24 (3.0%); and Sweden N=11 (1.8%). Other outcomes of interest including spontaneous preterm birth occurred in 3.6% (N=143) and SGA in 10.5% (N=416). Missing data was minimal (<1%) for key study outcomes, while there was ≥1% missing data for mode of delivery (1.0%) and Apgar score at 1 minute and 5 minutes (1.6% and 1.8%, respectively) ( Table 3).

**Table 3.** Number of outcomes in the IMPROvED cohort within 72 hours following delivery (N=3,951).

Outcome	N (%)
Preeclampsia	122 (3.1)
Gestational hypertension	179 (4.5)
PPROM	46 (1.2)
Placental abruption	14 (0.4)
Spontaneous preterm birth before 37 weeks’ gestation	143 (3.6)
Spontaneous preterm birth before 34 weeks’ gestation	34 (0.9)
Spontaneous preterm birth before 28 weeks’ gestation	9 (0.2)
SGA (birthweight <10th customised centile)	416 (10.5)
*Mode of delivery*	
Spontaneous vaginal delivery	1973 (49.9)
Operative vaginal delivery	1003 (25.4)
Prelabour caesarean section	361 (9.1)
Emergency caesarean section	577 (14.6)
Missing	37 (1.0)
*Apgar score 1 minute*	
7–10 (high)	3544 (89.7)
0–6 (low/intermediate)	342 (8.7)
Missing	65 (1.6)
*Apgar score 5 minutes*	
7–10 (high)	3795 (96.0)
0–6 (low/intermediate)	87 (2.2)
Missing	69 (1.8)
Any severe neonatal morbidity/mortality	32 (0.8)
Infant admitted to neonatal unit	404 (10.2)

Abbreviations: PPROM, preterm premature rupture of membranes; SGA, small for gestational age. If missing data ≥1%, n (%) reported for missing data.

## Discussion

### Conducted analyses

*ProTest:* The original protein biomarker study identified, verified, and validated novel panels of protein biomarkers for the prediction of preeclampsia at ~20 weeks’ gestation ^7^. From this study, a panel of five protein biomarkers was selected for development into a clinical assay. These proteins were: insulin-like growth factor acid labile subunit (IGFALS), serine peptidase inhibitor Kunitz type 1 (SPINT1), melanoma cell adhesion molecule (MCAM), and the angiogenic factors placental growth factor (PlGF) and soluble endoglin (sENG), by now well-established markers for preeclampsia ^10^. Throughout the protein biomarker study, various mass spectrometric techniques were applied to firstly identify ^11^ and then quantify the proteins of interest in a targeted fashion ^12, 13^. However, in 2012, mass spectrometry based multiplex protein analyses were not well established in clinical laboratory routine. A technology transfer from mass spectrometry based analyses to another multiplexing technology ^14^ compatible with antibody based immunoassay technologies for protein analyses was therefore deemed strategic for any future market acceptance of ProTest. As part of this transfer, specific antibody pairs for the proteins in the ProTest panel needed to be generated and evaluated for technical feasibility. With PlGF technology already available in the market, efforts were focused on developing a multiplex protein assay for the four other protein biomarkers. Unfortunately, severe technical issues were encountered. First, the IGFALS detection antibody cross reacted with capture antibodies against MCAM, SPINT1, and sENG, resulting in false positive results. Despite extensive further assay optimization efforts to resolve antibody cross-reactivity, the IGFALS assay could not be incorporated in a multiplex assay; a decision not to progress *de novo* antibody development for IGFALS was taken. Second, inadequate analytical sensitivity was obtained for SPINT1. Although dedicated assay parameter optimisation yielded sufficient analytical sensitivity, these parameters were not compatible with the parameters applicable for other protein assays, rendering SPINT analysis incompatible with multiplexing techniques. By that time, the clinical use case for a 20 weeks preeclampsia screening solution was put into question by meta-analyses indicating that aspirin prophylaxis to prevent (preterm) preeclampsia needed to start before <16 weeks’ gestation ^15, 16^, this finding was corroborated by the results of the ASPRE trial ^17^. Confronted with the multitude of technical hurdles and changes in screening test requirements, MyCartis (Belgium) developing ProTest abandoned the project.

### Ongoing analyses

*MetTest:* The original metabolite biomarker study used a single LC-MS experimental set-up to analyse deproteinised metabolite extracts from patient blood specimens (EDTA plasma) collected at ~15 weeks’ gestation in a discovery/validation metabolism profiling study. This resulted in a multivariate predictive model combining 14 putatively identified metabolites ^6^. In contrast to proteins, mass spectrometry based multiplex metabolite analyses were well established in clinical laboratory settings at the start of IMPROvED project; notably newborn screening for inborn errors of metabolism has a worldwide clinical application ^18, 19^. Hence, there was no commercial imperative to migrate the metabolite analyses to another analytical technology platform. Instead, the IMPROvED translational research planning for MetTest focused on converting the results of the metabolism profiling study into targeted LC-MS analyses based on the use of reference materials, as required for future application in clinical laboratories. Early in the IMPROvED project it was found that for many of the metabolite biomarkers in the original MetTest multivariate model no reference materials were readily available, thus the original metabolite panel was not amenable to further clinical and commercial development. In response, Metabolomic Diagnostics (Ireland) developing MetTest was compelled to establish an in-house LC-MS translational research workflow centring on multiplexing targeted LC-MS assays for 10s of putative metabolite biomarkers, whereby the availability of reference materials was a selection criterion for biomarker inclusion ^20^.

In parallel, MetTest researchers looked into formalising screening targets for preeclampsia screening in low-risk nulliparous women. This led to novel methodology to assess predictive values, statistics relevant to clinical practitioners, directly from receiver operating characteristic curves, used by test developers to summarise a test’s diagnostic performances in function of test sensitivity and test specificity ^21^. Using this methodology, it was proposed that a preeclampsia risk stratification test for nulliparous should ideally mimic the preeclampsia risk information as available for a second-time pregnant woman ^21^. The IMPROvED Consortium used these screening targets to perform an early cost-effectiveness analysis to assess both costs and health outcomes of a new screening test that would deliver such risk stratification ^22^.

During the lifetime of the IMPROvED project, PlGF gained wide-spread acceptance as an important preeclampsia risk biomarker, and early pregnancy preterm preeclampsia risk screening evolved into a separate clinical application. In response, MetTest was re-envisioned as a test that combined PlGF and metabolite biomarkers to deliver improved preterm preeclampsia as well as preeclampsia screening in nulliparous women. Using the purpose-developed LC-MS translational research workflow, candidate metabolite biomarkers were analysed in a case-control study. Models were evaluated in function of two pre-defined clinical use scenarios: (1) identify women at risk of developing preterm preeclampsia and (2) identify women at risk of developing preeclampsia at any stage of the pregnancy. It was found that combining dilinoleoyl-glycerol with PlGF effectively predicted increased preterm preeclampsia risk at ca. 15 weeks’ gestation. The further addition of heptadecanoyl-2-hydroxy-sn-glycero-3-phosphocholine expanded the capacity to also identify pregnant women at decreased risk of developing any form of preeclampsia ^20^.

In a further evolution of MetTest, it was shown that metabolite biomarkers can differentially predict preterm preeclampsia across body mass index classes ^23^, supporting the existence of distinct maternal risk profiles, a contemporary understanding in preeclampsia research ^24– 26^. Using machine learning methodology, these findings led to the development of novel prediction algorithms for preterm preeclampsia prediction in all pregnant women. Metabolite biomarkers augmented the established biomarkers PlGF, mean arterial pressure (MAP), and uterine artery pulsatility index (UTA-PI). Three novel prediction models were developed for three scenarios reflecting different levels of screening resources available; in each scenario use of metabolite biomarkers improved preterm preeclampsia prediction over the comparator models without metabolites. Classification of the pregnant women according to the maternal characteristics body mass index and/or race proved instrumental in achieving improved prediction ^27^. The latest iteration of MetTest is currently being developed into a clinical test.

### Publications

A recent publication ^28^ by members of the current study used IMPROvED data to examine the association between socioeconomic status and pregnancy and neonatal outcomes. We did not find strong evidence of associations between individual-level socioeconomic factors and pregnancy and neonatal outcomes overall, with only few significant associations observed among pregnancy outcomes. It is anticipated that IMPROvED data will be used in further maternal and child health secondary analysis research in the future.

Other publications arising as part of the IMPROvED Consortium include a systematic review and meta-analysis examining early pregnancy biomarkers in preeclampsia ^29^, as well as a cost-effectiveness analysis of screening for preeclampsia in nulliparous women ^22^.

### Strengths and limitations and further details

The IMPROvED Study has some limitations that should be noted. First, at time of enrolment, the majority of participants recruited had a third level of education, were in paid employment, and were married or in a stable relationship. Therefore, those with lower-level socioeconomic indicators may be underrepresented in the current cohort. Second, while recruitment was initially planned in five European countries ( *i.e.*, Republic of Ireland, United Kingdom, The Netherlands, Sweden, and Germany), the German site withdrew from the study at an early stage and were therefore not included in the IMPROvED cohort ^8^. Third, target recruitment numbers were not met resulting in 212 fewer participants than what was originally anticipated among participating countries. However, there was little data lost (~2%) between enrolment (N=4038) and the final study visit (N=3951) reducing the potential for selection bias driven by attrition from the cohort. Fourth, participants were not universally screened for the presence of gestational diabetes mellitus (GDM), potentially underestimating the incidence of GDM in the study. Finally, data on dietary behaviours was not collected. Poor dietary patterns, before and during pregnancy, have been shown to increase the likelihood of preeclampsia and may be an important risk factor to consider in future research ^30^.

There are also several strengths. First, IMPROvED contains both epidemiological data and biobank data that utilised numerous aliquots on multiple media enabling high quality research into the cause and prevention of adverse pregnancy outcomes. Specimens were collected at multiple time points throughout pregnancy to enhance the range of future research that can be conducted using the IMPROvED residual biobank. Second, all data were collected in a standardised manner by trained research midwives following detailed study specific standard operating procedures. This was to ensure standardised processes across recruitment centres to minimise bias during recruitment and data collection. Third, there was minimal missing data (<1%) among baseline characteristics and key outcomes, therefore maintaining the statistical power and representativeness of the cohort ^31^. Finally, this cohort profile increases awareness among the scientific community of the potential to access IMPROvED data and biobank samples. This, in turn, could foster collaborations and encourage researchers to obtain funding and ethical approval for evidence-based studies they would not normally be in a position to perform because of lack of access to large patient cohorts.

## Collaboration

The IMPROvED team encourages the use of the IMPROvED cohort data for research purposes. In supplying data, the IMPROvED team must comply with its obligations of confidentiality under the Data Protection Acts of 1988 and 2000 as well as with the General Data Protection Regulation (GDPR) of 2018. The use of the data by the applicant must also be consistent with these Acts and Regulations. Therefore, only requests for anonymised data will be considered. Data sharing on a public repository is prohibited.

## Data Availability

Researchers can apply to access IMPROvED epidemiological and biobank data by contacting IMPROvED Principal Investigator, Dr Fergus McCarthy, University College Cork, Ireland in the first instance ( fergus.mccarthy@ucc.ie), followed by submission of a proposal to the IMPROvED Consortium. The data are not publicly available due to privacy/ethical restrictions and only available upon reasonable request. For further information, please email Dr Gillian Maher at gillian.maher@ucc.ie.

## References

[1] Magee LA Brown MA Hall DR : The 2021 International Society for the Study of Hypertension in Pregnancy classification, diagnosis & management recommendations for international practice. * Pregnancy Hypertens.* 2022;27:148–69. 10.1016/j.preghy.2021.09.008 35066406

[2] Navaratnam K Alfirevic Z Baker PN : A multi-centre phase IIa clinical study of predictive testing for preeclampsia: improved pregnancy outcomes via early detection (IMPROvED). *BMC Pregnancy Childbirth.* 2013;13(1): 226. 10.1186/1471-2393-13-226 24314209 PMC4029471

[3] Rana S Lemoine E Granger J : Preeclampsia. *Circ Res.* 2019;124(7):1094–112. 30920918 10.1161/CIRCRESAHA.118.313276

[4] Barrett PM McCarthy FP Kublickiene K : Adverse Pregnancy Outcomes and Long-term Maternal Kidney Disease: A Systematic Review and Meta-analysis. *JAMA Netw Open.* 2020;3(2): e1920964. 10.1001/jamanetworkopen.2019.20964 32049292 PMC12527481

[5] Leon LJ McCarthy FP Direk K : Preeclampsia and Cardiovascular Disease in a Large UK Pregnancy Cohort of Linked Electronic Health Records: A CALIBER Study. *Circulation.* 2019;140(13):1050–60. 10.1161/CIRCULATIONAHA.118.038080 31545680

[6] Kenny LC Broadhurst DI Dunn W : Robust early pregnancy prediction of later preeclampsia using metabolomic biomarkers. *Hypertension.* 2010;56(4):741–9. 10.1161/HYPERTENSIONAHA.110.157297 20837882 PMC7614124

[7] Myers JE Tuytten R Thomas G : Integrated proteomics pipeline yields novel biomarkers for predicting preeclampsia. *Hypertension.* 2013;61(6):1281–8. 10.1161/HYPERTENSIONAHA.113.01168 23547239

[8] CORDIS EU Research Results: IMPROvED: Improved Pregnancy Outcomes by Early Detection; personalized medicine for pregnant women: novel metabolomic and proteomic biomarkers to detect pre-eclampsia and improve outcome. 2018; [cited 2023 6th July]. Reference Source

[9] Umesawa M Kobashi G : Epidemiology of hypertensive disorders in pregnancy: prevalence, risk factors, predictors and prognosis. *Hypertens Res.* 2017;40(3):213–20. 10.1038/hr.2016.126 27682655

[10] Cerdeira AS Agrawal S Staff AC : Angiogenic factors: potential to change clinical practice in pre-eclampsia? *BJOG.* 2018;125(11):1389–95. 10.1111/1471-0528.15042 29193681 PMC6175139

[11] Staes A Impens F Van Damme P : Selecting protein N-terminal peptides by combined fractional diagonal chromatography. *Nat Protoc.* 2011;6(8):1130–41. 10.1038/nprot.2011.355 21799483

[12] Rifai N Gillette MA Carr SA : Protein biomarker discovery and validation: the long and uncertain path to clinical utility. *Nat Biotechnol.* 2006;24(8):971–83. 10.1038/nbt1235 16900146

[13] Carr SA Abbatiello SE Ackermann BL : Targeted peptide measurements in biology and medicine: best practices for mass spectrometry-based assay development using a fit-for-purpose approach. *Mol Cell Proteomics.* 2014;13(3):907–17. 10.1074/mcp.M113.036095 24443746 PMC3945918

[14] Falconnet D She J Tornay R : Rapid, sensitive and real-time multiplexing platform for the analysis of protein and nucleic-acid biomarkers. *Anal Chem.* 2015;87(3):1582–9. 10.1021/ac502741c 25567587

[15] Roberge S Villa P Nicolaides K : Early administration of low-dose aspirin for the prevention of preterm and term preeclampsia: a systematic review and meta-analysis. *Fetal Diagn Ther.* 2012;31(3):141–6. 10.1159/000336662 22441437

[16] Bujold E Roberge S Lacasse Y : Prevention of preeclampsia and intrauterine growth restriction with aspirin started in early pregnancy: a meta-analysis. *Obstet Gynecol.* 2010;116(2 Pt 1):402–14. 10.1097/AOG.0b013e3181e9322a 20664402

[17] Rolnik DL Wright D Poon LC : Aspirin versus Placebo in Pregnancies at High Risk for Preterm Preeclampsia. *N Engl J Med.* 2017;377(7):613–22. 10.1056/NEJMoa1704559 28657417

[18] François R Yves G Marie-Thérèse B : Newborn Screening By Tandem Mass Spectrometry: Impacts, Implications and Perspectives.In: Jeevan KP, editor. *Tandem Mass Spectrometry*. Rijeka: IntechOpen;2012;31. 10.5772/33118

[19] Therrell BL Padilla CD Loeber JG : Current status of newborn screening worldwide: 2015. *Semin Perinatol.* 2015;39(3):171–87. 10.1053/j.semperi.2015.03.002 25979780

[20] Kenny LC Thomas G Poston L : Prediction of preeclampsia risk in first time pregnant women: Metabolite biomarkers for a clinical test. *PLoS One.* 2020;15(12): e0244369. 10.1371/journal.pone.0244369 33370367 PMC7769282

[21] Thomas G Kenny LC Baker PN : A novel method for interrogating receiver operating characteristic curves for assessing prognostic tests. *Diagn Progn Res.* 2017;1: 17. 10.1186/s41512-017-0017-y 31093546 PMC6460848

[22] Zakiyah N Tuytten R Baker PN : Early cost-effectiveness analysis of screening for preeclampsia in nulliparous women: a modelling approach in European high-income settings. *PLoS One.* 2022;17(4): e0267313. 10.1371/journal.pone.0267313 35446907 PMC9022877

[23] Tuytten R Syngelaki A Thomas G : First-trimester preterm preeclampsia prediction with metabolite biomarkers: differential prediction according to maternal body mass index. *Am J Obstet Gynecol.* 2023;229(1):55.e1–55.e10. 10.1016/j.ajog.2022.12.012 36539025

[24] Roberts JM Rich-Edwards JW McElrath TF : Subtypes of preeclampsia: recognition and determining clinical usefulness. *Hypertension.* 2021;77(5):1430–41. 10.1161/HYPERTENSIONAHA.120.14781 33775113 PMC8103569

[25] Than NG Romero R Tarca AL : Integrated systems biology approach identifies novel maternal and placental pathways of preeclampsia. *Front Immunol.* 2018;9: 1661. 10.3389/fimmu.2018.01661 30135684 PMC6092567

[26] Than NG Posta M Györffy D : Early pathways, biomarkers, and four distinct molecular subclasses of preeclampsia: the intersection of clinical, pathological, and high-dimensional biology studies. *Placenta.* 2022;125:10–9. 10.1016/j.placenta.2022.03.009 35428514 PMC9261837

[27] Thomas G Syngelaki A Hamed K : Preterm preeclampsia screening using biomarkers: combining phenotypic classifiers into robust prediction models. *Am J Obstet Gynecol MFM.* 2023;5(10): 101110. 10.1016/j.ajogmf.2023.101110 37752025

[28] Maher GM Ward LJ Hernandez L : Association between socioeconomic status with pregnancy and neonatal outcomes: An international multicenter cohort. *Acta Obstet Gynecol Scand.* 2023;102(11):1459–1468. 10.1111/aogs.14659 37602747 PMC10577636

[29] Wu P Van den Berg C Alfirevic Z : Early pregnancy biomarkers in pre-eclampsia: a systematic review and meta-analysis. *Int J Mol Sci.* 2015;16(9):23035–56. 10.3390/ijms160923035 26404264 PMC4613350

[30] Esquivel MK : Nutritional Status and Nutrients Related to Pre-Eclampsia Risk. * Am J Lifestyle Med.* 2023;17(1):41–5. 10.1177/15598276221129841 36636396 PMC9830236

[31] Kang H : The prevention and handling of the missing data. *Korean J Anesthesiol.* 2013;64(5):402–6. 10.4097/kjae.2013.64.5.402 23741561 PMC3668100

