# Supplementary material for: Cohort profile: Improved Pregnancy Outcomes via Early Detection (IMPROvED), an International Multicentre Prospective Cohort
Source: HRB Open Res. 2024 Jun 24;6:65. Originally published 2023 Nov 13. [Version 3] doi: 10.12688/hrbopenres.13812.3 (PMC11190647; doi:10.12688/hrbopenres.13812.3)
Supplement: Peer review history

# Reviewer report: Reviewer response for version 3

**DOI:** 10.21956/hrbopenres.15275.r44034

**Published:** 2024-12-27

**Authors:** Jimmy Espinoza

**Affiliations:** 1 University of Texas Health Science Center Houston, Houston, USA

The authors report the results on a large ongoing longitudinal multicenter cohort study aimed at an early identification of clinical and laboratory parameters associated with adverse pregnancy outcomes including preeclampsia, fetal growth restriction, and preterm delivery. The authors tested the role of a combination of several biomarkers including placental growth factor (PlGF) and others in the identification of individuals at increased risk for complications; however, the authors encountered several hurdles in this process and their work is ongoing. During the study period the authors created a detailed database and biorepository of maternal samples of blood, urine, placenta, umbilical cord, and hair collected at least at two different gestational ages as well as fetal blood that could be available to other researchers willing to collaborate with the authors. They should be congratulated in their efforts

 Additional comments:

 - There is a solid body of evidence supporting the use maternal plasma or serum PlGF, Doppler evaluation of the uterine arteries, mean maternal blood pressure and other maternal characteristics in the evaluation of risk for pregnancy complications. The authors are encouraged to further validate these observations. Although this strategy was used to evaluate the risk for preeclampsia, it has also been proposed to evaluate the risk for other pregnancy complications

 - If the panels of protein biomarkers at 15 weeks and 20 weeks’ gestation for preeclampsia risk assessment were previously validated, why did the commercial entities used by the authors encountered technical issue that precluded the use of these panels?

Is the work clearly and accurately presented and does it cite the current literature?

Partly

If applicable, is the statistical analysis and its interpretation appropriate?

I cannot comment. A qualified statistician is required.

Are all the source data underlying the results available to ensure full reproducibility?

No source data required

Is the study design appropriate and is the work technically sound?

Yes

Are the conclusions drawn adequately supported by the results?

Partly

Are sufficient details of methods and analysis provided to allow replication by others?

Yes

Reviewer Expertise:

Preeclampsia, fetal growth restriction, fetal Doppler evaluation

I confirm that I have read this submission and believe that I have an appropriate level of expertise to confirm that it is of an acceptable scientific standard, however I have significant reservations, as outlined above.

# Reviewer report: Reviewer response for version 3

**DOI:** 10.21956/hrbopenres.15275.r44035

**Published:** 2024-12-26

**Authors:** Khanisyah Erza Gumilar

**Affiliations:** 1 Universitas Airlangga, Surabaya, East Java, Indonesia

This paper describes the IMPROvED study, which aims to develop a clinical test for detecting the risk of preeclampsia during pregnancy. This multicenter phase IIa study collects demographic, clinical, and biobank data from 4,038 low-risk nulliparous singleton pregnancies recruited from maternity units in Ireland, the United Kingdom, the Netherlands, and Sweden. Data is obtained through interviews and biobank samples (blood, urine, and hair) collected throughout pregnancy to develop an accurate and reliable preeclampsia risk assessment test.

**Introduction**

 In this chapter, the author effectively explains the background of the study and the importance of developing a screening test for preeclampsia. Have previous studies attempted to develop a similar screening test? How does this study differ from or improve upon previous research?

**Methods**

 This prospective study uses sampling at multiple pregnancy periods, providing deep insights into the development of biomarkers over time. Data quality is ensured through well-established protocols and data collection by trained healthcare professionals. However, there are some limitations:

- The narrow inclusion criteria, such as focusing only on low-risk singleton pregnancies, limit the generalizability of the results to high-risk populations, such as multiparous pregnancies.
- The study has not achieved its initial recruitment target (one site in Germany withdrew from the study).

Questions:

- How can the findings of this study be applied to a more heterogeneous high-risk population?
- Are there efforts to address the lack of participation from low socioeconomic groups? Is there an analysis of potential bias due to the withdrawal of the German site?

**Results**

 This chapter is presented clearly, including participants' characteristics and key findings related to preeclampsia and other pregnancy outcomes. The data loss rate is very low (<1%), which reinforces the credibility of the results. The clinical and biobank data collected offer significant opportunities for further research, such as the development of predictive algorithms. However, there is still a point to be highlighted. The lack of representation of ethnicities other than Northern Europeans reduces the relevance to the global population. What strategic steps can be taken to expand ethnic representation in future research?

**Discussion:**

 The discussion effectively summarizes the analyses conducted (ProTest and MetTest) and explains the challenges encountered in the development of the tests. Are there plans to continue the development of ProTest in the future, or is the focus solely on MetTest?

Is the work clearly and accurately presented and does it cite the current literature?

Yes

If applicable, is the statistical analysis and its interpretation appropriate?

Yes

Are all the source data underlying the results available to ensure full reproducibility?

Yes

Is the study design appropriate and is the work technically sound?

Yes

Are the conclusions drawn adequately supported by the results?

Yes

Are sufficient details of methods and analysis provided to allow replication by others?

Yes

Reviewer Expertise:

Preeclampsia

I confirm that I have read this submission and believe that I have an appropriate level of expertise to confirm that it is of an acceptable scientific standard, however I have significant reservations, as outlined above.

# Reviewer report: Reviewer response for version 2

**DOI:** 10.21956/hrbopenres.15203.r39337

**Published:** 2024-06-20

**Authors:** Shaun Brennecke

**Affiliations:** 1 Department of Obstetrics and Gynaecology, The University of Melbourne, Melbourne, Victoria, Australia

This report summarises the history, aims, content and uses-to-date of the IMPROVeD cohort, a collection of clinical data and measurements, together with various biobanked samples, from 4000 or so pregnant women.

 The cohort was established mostly with a view to enabling the development of biomarkers for the prediction of preeclampsia.

 This goal seems to have met with mixed success, and now the consortium of researchers responsible for the cohort is altruistically offering it for others to potentially take advantage of its rich store of data and samples.

 The report itself is therefore descriptive rather than analytic in nature, and amounts to an extended and detailed invitation to potential research collaborators to consider using this large and valuable cohort to progress knowledge relating to the aetiology, prevention and improved management of pregnancy complications.

 As such, the usual criteria for judging the scientific merits of an academic journal submission describing the results of a clinical study or laboratory investigation are not especially applicable to this article.

 Depending on their particular needs, external researchers interested in accepting the invitation to collaborate will need to make their own individualised assessments of the potential utility of the cohort's available data and samples.

 A principle of *caveat emptor* therefore applies in situations such as this, with notice being taken of the relevance or otherwise of the patient inclusion and exclusion criteria, stages of pregnancy at which data and samples were obtained, the type of data and samples that were collected, the standard operating procedures and level of quality control for sample collection, processing, aliquotting, and storage, and so on.

 For example, the range of countries contributing to the cohort suggests the ethnicity/racial background of the women included in it is likely to be mostly northern European. Analyses of the cohort may therefore not be as informative when applied to other, different ethnicities/racial backgrounds.

 As well, the cohort's relatively narrow inclusion and rather broad exclusion criteria mean extrapolation of cohort analyses to other real-world pregnancy situations (multiparous women, multiple pregnancies, women with relevant comorbidities such as chronic hypertension, diabetes, SLE, CKD, APL syndrome, etc) is likely to be limited.

 That said, as has been the case with other similarly available large collections, subsequent, post hoc, retrospective interrogations by external collaborators can prove of value in various ways, including by providing suggestive and/or preliminary data for further more definitive prospective studies.

 Of particular benefit within the IMPROVeD cohort are the three sampling points at previable stages of pregnancy (late first/early-to-mid second timester). They provide ample scope for the determination of predictive algorithms and tests at a sufficiently early moment in a pregnancy to be not only of likely clinical value for prediction and institution of prophylactic interventions, but also of practical utility with respect to patient availability, given most pregnant women in countries with similar maternity care environments to the those providing the cohort will have attended for care by then.

 This benefit is enhanced by the impressivley large size of the IMPROVeD cohort.

 In terms of the report itself, as a description of the IMPROVeD cohort and as an invitation to potential  collaborators to consider availing themselves of its wealth of data and samples, it meets these aims well enough.

 Notwithstanding the above comments, the only suggestions I offer for changing the manuscript are to update reference 1 to the latest ISSHP pronouncement on the classification etc of the hypertensive disorders of pregnancy (see  PMID: 35066406) and to correct the presumed error in Table 2 in the Biobank section wherein placental samples are purported to have been taken at 11 and 15 weeks' gestation, rather than at birth/shortly after delivery (as stated in the main body of the article on page 5).

Is the work clearly and accurately presented and does it cite the current literature?

Yes

If applicable, is the statistical analysis and its interpretation appropriate?

Not applicable

Are all the source data underlying the results available to ensure full reproducibility?

Yes

Is the study design appropriate and is the work technically sound?

Yes

Are the conclusions drawn adequately supported by the results?

Yes

Are sufficient details of methods and analysis provided to allow replication by others?

Yes

Reviewer Expertise:

Preeclampsia and other complications of human pregnancy.

I confirm that I have read this submission and believe that I have an appropriate level of expertise to confirm that it is of an acceptable scientific standard.

# Response

**Published:** 2024-06-21

**Authors:** Gillian Maher

**Affiliations:** University College Cork, Ireland

Dear Prof Brennecke,

 We thank you for your detailed summary and helpful review of our manuscript entitled *“Cohort profile: Improved Pregnancy Outcomes via Early Detection (IMPROvED), an International Multicentre Prospective Cohort”*.

 Please find below an italicized reply addressing each suggested change in your Reviewer’s Report.

*1.* *Update reference 1 to the latest ISSHP pronouncement on the classification etc of the hypertensive disorders of pregnancy (see  PMID: 35066406).*

               We have now updated reference one in our manuscript to ISSHP’s latest classification, diagnosis, and management recommendations of hypertensive disorders of pregnancy:

 Magee LA, Brown MA, Hall DR, Gupte S, Hennessy A, Karumanchi SA, et al. The 2021 International Society for the Study of Hypertension in Pregnancy classification, diagnosis & management recommendations for international practice. Pregnancy hypertension. 2022;27:148-69. 10.1016/j.preghy.2021.09.008

 2. *Correct the presumed error in Table 2 in the Biobank section wherein placental samples are purported to have been taken at 11 and 15 weeks' gestation, rather than at birth/shortly after delivery (as stated in the main body of the article on page 5).*

 We apologise for this error that occurred during typesetting and we thank you for bringing it to our attention. We have now corrected the error in Table 2 to illustrate that placental samples were taken at birth.

 We hope these amendments address the suggestions raised. Should any further amendments be necessary, we would be happy to address them accordingly. 

 Sincerely,

 Dr Gillian Maher, Prof Louise Kenny, Dr Kate Navaratnam, Prof Zarko Alfirevic, Dr Darina Sheehan, Prof Philip Baker, Prof Christian Gluud, Dr Robin Tuytten, Prof Marius Kublickas, Dr Boel Niklasson, Prof Johannes Duvekot, Prof Caroline van den Berg, Prof Pensee Wu, Prof Karolina Kublickiene, Dr Fergus McCarthy, and Prof Ali Khashan

# Reviewer report: Reviewer response for version 2

**DOI:** 10.21956/hrbopenres.15203.r38190

**Published:** 2024-03-04

**Authors:** Hongmei Li

**Affiliations:** 1 Department of Epidemiology and Biostatistics, School of Public Health, Suzhou Medical College, Soochow University, Suzhou, China

Authors have answered my questions, and I have no other  comments.

Is the work clearly and accurately presented and does it cite the current literature?

Yes

If applicable, is the statistical analysis and its interpretation appropriate?

Yes

Are all the source data underlying the results available to ensure full reproducibility?

Yes

Is the study design appropriate and is the work technically sound?

Yes

Are the conclusions drawn adequately supported by the results?

Yes

Are sufficient details of methods and analysis provided to allow replication by others?

Yes

Reviewer Expertise:

hypertensive disorders in pregnancy

I confirm that I have read this submission and believe that I have an appropriate level of expertise to confirm that it is of an acceptable scientific standard.

# Reviewer report: Reviewer response for version 1

**DOI:** 10.21956/hrbopenres.15116.r37795

**Published:** 2024-01-30

**Authors:** Hongmei Li

**Affiliations:** 1 Department of Epidemiology and Biostatistics, School of Public Health, Suzhou Medical College, Soochow University, Suzhou, China

This article provided a description for the profile including existing research results of IMPROvED cohort study. Overall, the cohort study was well designed. It is expected that this cohort study will have exciting results in the risk assessment for preeclampsia.

 However, I have some questions:

1. First, for this research design, what is the reason for a short time interval between the first collection of specimens at 9-13 gestational weeks and the second collection at 14-16 gestational weeks ? Of the 4038 participants, only 1076 completed the first sample collection. What was the impact on the overall study?
2. Second, the dietary pattern is also a contributing factor to preeclampsia, and the dietary habits vary among different countries. Whether dietary behaviors were considered for collection when designing this cohort study? If not, it is recommended to discuss in the limitation section.

Is the work clearly and accurately presented and does it cite the current literature?

Yes

If applicable, is the statistical analysis and its interpretation appropriate?

Yes

Are all the source data underlying the results available to ensure full reproducibility?

Yes

Is the study design appropriate and is the work technically sound?

Yes

Are the conclusions drawn adequately supported by the results?

Yes

Are sufficient details of methods and analysis provided to allow replication by others?

Yes

Reviewer Expertise:

hypertensive disorders in pregnancy

I confirm that I have read this submission and believe that I have an appropriate level of expertise to confirm that it is of an acceptable scientific standard, however I have significant reservations, as outlined above.

# Response

**Published:** 2024-02-01

**Authors:** Gillian Maher

**Affiliations:** University College Cork, Ireland

Dear Dr Hongmei Li,

 We thank you for your helpful review of our manuscript entitled *“Cohort profile: Improved Pregnancy Outcomes via Early Detection (IMPROvED), an International Multicentre Prospective Cohort”*.

 Please find below an itemized reply addressing each comment in your Reviewer’s Report.

*1.* ***This article provided a description for the profile including existing research results of IMPROvED cohort study. Overall, the cohort study was well designed. It is expected that this cohort study will have exciting results in the risk assessment for preeclampsia.***

***However, I have some questions:***

***First, for this research design, what is the reason for a short time interval between the first collection of specimens at 9-13 gestational weeks and the second collection at 14-16 gestational weeks ? Of the 4038 participants, only 1076 completed the first sample collection. What was the impact on the overall study?***

**Response :** We thank you for your positive comment regarding the IMPROvED cohort study.

 Specimens were collected at four time points throughout pregnancy to enhance the range of future research that can be conducted using IMPROvED biobank data. We have edited our Discussion to include this information as follows:

 “IMPROvED contains both epidemiological data and biobank data that utilised numerous aliquots on multiple media enabling high quality research into the cause and prevention of adverse pregnancy outcomes. Specimens were collected at multiple time points throughout pregnancy to enhance the range of future research that can be conducted using the IMPROvED residual biobank.”

 The first study visit at 11+0 to 13+6 weeks’ gestation was optional. Therefore, while participation at this first time-point was desirable, it was not mandatory, resulting in fewer completed samples than the second (mandatory) study visit (~15 weeks’ gestation). This information is outlined in our Methods under ‘Data collection and follow-up’.

 2. ***Second, the dietary pattern is also a contributing factor to preeclampsia, and the dietary habits vary among different countries. Whether dietary behaviours were considered for collection when designing this cohort study? If not, it is recommended to discuss in the limitation section.***

**Response:** We agree that poor dietary pattern is an important risk factor for preeclampsia. However, the IMPROvED cohort study did not collect data on dietary behaviours. We have edited our Limitations to include your suggestion, as follows:

 “Data on dietary behaviours was not collected. Poor dietary patterns, before and during pregnancy, have been shown to increase the likelihood of preeclampsia and may be an important risk factor to consider in future research (1).”

 We hope these amendments address the suggestions raised. Should any further amendments be necessary, we would be happy to address them accordingly. 

 Sincerely,

 Dr Gillian Maher, Prof Louise Kenny, Dr Kate Navaratnam, Prof Zarko Alfirevic, Dr Darina Sheehan, Prof Philip Baker, Prof Christian Gluud, Dr Robin Tuytten, Prof Marius Kublickas, Dr Boel Niklasson, Prof Johannes Duvekot, Prof Caroline van den Berg, Prof Pensee Wu, Prof Karolina Kublickiene, Dr Fergus McCarthy, and Dr Ali Khashan

**Reference**

 1.  Esquivel MK. Nutritional Status and Nutrients Related to Pre-Eclampsia Risk. American journal of lifestyle medicine. 2023;17(1):41-5.
